# Optimization of regeneration using differential growth regulators in indica rice cultivars

**DOI:** 10.1007/s13205-015-0343-0

**Published:** 2016-01-09

**Authors:** Samar Shekar R. Sankepally, Bharat Singh

**Affiliations:** Institute of Biotechnology, Amity University Rajasthan, Jaipur, 303002 India

**Keywords:** Micropropagation, Plantlets, *Oryza sativa*, Sambha mahsuri, Acclimatization

## Abstract

Paddy is a staple crop and being grown in major parts of India, but similarly it is facing some major challenges like abiotic stresses (drought, salinity, etc.) by which its productivity is greatly affected. De-husked seeds of 21 cultivars of indica rice were cultured onto MS medium for screening of their micropropagation capabilities. All the selected cultivars are being cultivated by farmers of Andhra Pradesh and Rajasthan states of India, these cultivars bear good quality of grain and high yield. Similarly, both states are facing the problem of drought so; we selected these cultivars for the micropropagation and transformation studies. The induction of callus and regeneration in these selected cultivars were achieved on MS culture medium with supplementation of various growth regulators. Maximum callus induction and regeneration frequency were observed in Sambha mahsuri at the concentration of 3.0 mg L^−1^ of 2,4-D and BAP (5.0 mg L^−1^) while the moderate response achieved in other cultivars of rice. Sambha mahsuri showed promising results in terms of callus induction frequency, and regeneration potentialities of embryogenic callus so, there is need to develop a rice cultivars that could thrive under drought condition if the country is to sustain rice production. Finally, regenerated plantlets were successfully transferred to natural conditions (in pots) after acclimatization.

## Introduction

*Oryza sativa* L. is the most economically important cultivated rice species of the Oryzeae tribe of Poaceae family. Rice is the world’s single most important staple food crop, and it represents a primary food for more than one-third of its population (Ilahi et al. 2005). Tissue culture techniques have become necessary for the production of the transgenic rice plants (Peng et al. 1992), hybrids (Mariam et al. 1996), and for the recovery of germplasm when seed availability is limited. These methods are based on adventitious shoot culture (Shahsavari 2011) and somatic embryogenesis (Amarasinghe 2009) which resulted into genetic variations in rice cultivars (Mannan et al. 2013). Micropropagation protocols have been established for *japonica* and indica rice cultivars using as explants like as mature seeds (Ullah et al. 2007), shoot segments (Verma et al. 2011), embryos (Ali et al. 2004; Evangelista et al. 2009), anthers (Khatun et al. 2010) which produced regenerated plantlets. Fifteen rice cultivars of indica rice were screened for induction of callus as well as subsequent regeneration of plantlets through plant tissue culture (Hertke and Lörz 1989). Similarly, 500 cultivars of rice were comprehensively studied for the induction of callus as well as regeneration of plantlets by Kamia et al. (1988). It was also reported that an individual genotype of rice plays a significant role in the induction of callus and regeneration of plantlets (Hoque and Mansfield 2004; Islam et al. 2005). Sambha mahsuri, Cotton Dora sannalu, PR-115 and PR-116 are high yielding cultivars in Telangana, Andhra Pradesh and Rajasthan states of India so, we focused our study on these cultivars of indica rice.

## Materials and methods

### Plant material

The mature seeds of six cultivars (Sarsu 52, P-44, PR-116, PR-115, PAV-16, PAV-201) of indica rice were obtained (Aug, 2013) from State Farms Corporation of India (SFCI), Suratgarh, India and were authenticated by Neeraj Verma, Director-Incharge (SFCI), Suratgarh, India. The remaining 15 indica rice cultivars (Surekha, Bhadrakali, Erramallellu, Kavya, Warangal sambha, Ramappa, Warangal sannalu, Sheetal, Siddhi, Varalu, Khushava, Pothana, Cotton dora sannalu, Vijetha, Sambha mahsuri) obtained (Aug, 2013) from Agricultural Research Station (ARS), Warangal, India. The seeds of these 15 cultivars were authenticated by Dr. C. Cheralu, Associate Director, ARS, Warangal, India. The seeds of all 21 indica rice cultivars were stored at room temperature in plant tissue culture lab to keep them away from contamination.

### General experimental conditions

The authors designed the general experimental conditions as follows: (a) conditions for callus induction—each concentration of growth regulators, authors kept two replicas and five culture tubes for each concentration, i.e., ten tubes for each growth regulator concentration studied and one control for each concentration without addition of growth regulators; (b) multiple shoot formation—for each concentration of growth regulator, kept two replicas and five culture tubes for each concentration, i.e., ten tubes for each growth regulator concentration studied and one control for each concentration without addition of growth regulators; (c) profuse rooting—for each concentration of growth regulator, kept two replicas and five culture tubes for each concentration, i.e., ten tubes for each growth regulator concentration studied and one control for each concentration without addition of growth regulators.

### Culture conditions

De-husked seeds of the indica rice cultivars were washed with tap water to remove dust and other particles. The cleaned de-husked seeds were placed in 70 % alcohol for 2.0 min and surface sterilized with 1.0 % (w/v) mercuric chloride solution for 3.0 min. The treated seeds were then rinsed three times with sterilized distilled water to remove all the traces of mercuric chloride. The sterilized seeds were inoculated onto MS (Murashige and Skoog 1962) culture medium containing sucrose (30 g L^−1^) and supplemented with various concentrations with various combinations of 2,4-D (1.0–5.0 mg L^−1^), kinetin (1.0–5.0 mg L^−1^), BAP (1.0–5.0 mg L^−1^), IAA (1.0–5.0 mg L^−1^), NAA (1.0–3.0 mg L^−1^), IBA (0.0–1.0 mg L^−1^). These inoculated cultures were incubated at 25 ± 1 °C with 60 % relative humidity under room light conditions (300 Lux). Obtained callus, multiple shoots and shoots with profuse rooting were subcultured after 35 days onto fresh MS medium containing same concentration of growth regulators.

## Results

### Callus induction and proliferation

**Fig. 1:**
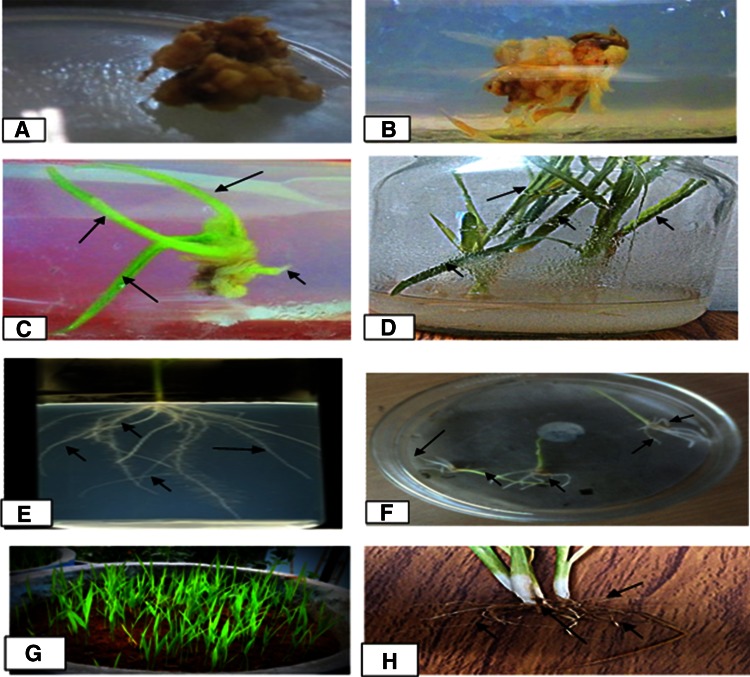
Micropropagation of Sambha mahsuri indica rice cultivar. **a**, **b** Callus induction and proliferation; **c**, **d** multiple shoot regeneration: **e**, **f**, profuse rooting; **g** plantlets transferred in pot; **h** roots of plantlet. *Arrows showing number of shoots and roots in their respective figures

Sterilized de-husked seeds of 21 indica rice cultivars were inoculated onto MS culture medium supplemented with different concentrations of growth regulators and 3.0 % sucrose. The used growth regulators were kinetin (2.5 mg L^−1^), 2,4-D (3.0 mg L^−1^), kinetin + 2,4-D (2.5 + 3.5 mg L^−1^), BAP + IAA (2.5 + 3.0 mg L^−1^) added to the MS basal medium. After about 10 days of inoculation, callus formation started in 11 cultivars (Sarsu-52, PR-115, PR-116, Erramallellu, Pothana, Vijetha, Surekha, Kavya, Sambha mahsuri, Ramappa, and Khusava) of indica rice (Table 1) while in the above mentioned doses of growth regulators, no callus was observed in the remaining 10 cultivars (P-44, PAV-16, PAV-201, Bhadrakali, Cotton dora Sannalu, Varalu, Sheetal, Siddhi, Warangal Sambha, Warangal Sanalu) of indica rice. The excellent creamish colored embryoids (Fig. 1) were achieved in Sambha mahsuri cultivar with 3.0 mg L^−1^ of 2,4-D supplement in MS culture medium. A soft, friable, brown colored callus with moderate growth was induced and proliferated in PR-116 with 3.0 mg L^−1^ 2,4-D supplement (Table 1). Besides Sambha mahsuri and PR-116 cultivar, embryogenic calli with mild growth were also observed in PR-115 and Erramallelu cultivars of indica rice. Non-embryogenic callus formed in Sarsu-52 cultivar of indica rice at the dose of 3.0 mg L^−1^ of 2,4-D (Table 1). There were some variations in calli also observed among all the 21 cultivars of indica rice qualitatively, i.e., size and color of calli, growth of callus. The observed variations in induction and proliferation of callus in different cultivars seemed to be mainly due to the physical factors like as position and location of explants onto the culture medium because of the same genetic make-up of explants. As evident from the observed results, meristemoids developed in these calli which exhibited sufficient reasonable regeneration capability. The 2,4-D supplementation in MS culture medium hastened the induction of callus in 11 cultivars of indica rice individually as well as in combination with cytokinins. Similarly, the effects of cytokinins (kinetin, BAP) in induction of calli were also observed in 11 cultivars of indica rice (Table 1). It was also observed that kinetin 2.5 mg L^−1^ was more effective than 2.5 + 3.0 mg L^−1^ BAP + IAA in induction and proliferation of calli in PR-115, PR-116 and Erramallelu, Pothana, Vijetha, Kavya, Sambha mahsuri, Rammapa, Khushava. When the concentration of kinetin was further increased to the level of 1.0 mg L^−1^, i.e., from 2.5 to 3.5 mg L^−1^ keeping the concentration of IAA as stable (2.5 mg L^−1^), excellent (e.g., weight) callus induction and growth was observed within 3–4 weeks in Sambha mahsuri, Erramallelu, Pothana (Table 1).

**Table 1:** Induction and characters of callus regenerated from de-husked seeds of indica rice cultivars cultured on MS culture medium with various concentrations of growth regulators

Name of the cultivars	Average response (%)^a^	Growth regulators used	Concentration of growth regulators (mg L^−1^)	Number of days taken
Sarsu-52	70.34 ± 0.39	KIN	2.5	28
	75.16 ± 0.48	2,4-D	3.0	27
	69.34 ± 0.17	KIN + 2,4-D	2.5 + 3.5	29
	65.23 ± 0.28	IAA + BAP	3.0 + 2.5	30
	70.66 ± 0.36	KIN + IAA	2.5 + 2.5	29
PR-115	73.45 ± 0.46	KIN	2.5	29
	75.55 ± 0.46	2,4-D	3.0	28
	70.66 ± 0.24	KIN + 2,4-D	2.5 + 3.5	29
	72.39 ± 0.15	IAA + BAP	3.0 + 2.5	30
	78.59 ± 0.81	KIN + IAA	2.5 + 2.5	29
PR-116	76.43 ± 0.72	KIN	2.5	28
	65.64 ± 0.63	2,4-D	3.0	32
	64.09 ± 0.63	KIN + 2,4-D	2.5 + 3.5	30
	70.72 ± 0.53	IAA + BAP	3.0 + 2.5	27
	72.65 ± 0.43	KIN + IAA	2.5 + 2.5	30
Erramallelu	70.89 ± 0.31	KIN	2.5	28
	69.58 ± 0.28	2,4-D	3.0	26
	71.67 ± 0.59	KIN + 2,4-D	2.5 + 3.5	27
	67.56 ± 0.60	IAA + BAP	3.0 + 2.5	27
	65.38 ± 0.73	KIN + IAA	2.5 + 2.5	28
Pothana	74.34 ± 0.61	KIN	2.5	30
	71.69 ± 0.41	2,4-D	3.0	32
	72.59 ± 0.46	KIN + 2,4-D	2.5 + 3.5	30
	68.41 ± 0.53	IAA + BAP	3.0 + 2.5	31
	70.27 ± 0.55	KIN + IAA	2.5 + 2.5	30
Vijetha	70.40 ± 0.50	KIN	2.5	32
	67.71 ± 0.49	2,4-D	3.0	31
	70.44 ± 0.47	KIN + 2,4-D	2.5 + 3.5	30
	65.35 ± 0.34	IAA + BAP	3.0 + 2.5	27
	58.78 ± 0.33	KIN + IAA	2.5 + 2.5	29
Surekha	64.45 ± 0.11	KIN	2.5	29
	64.51 ± 0.22	2,4-D	3.0	29
	65.38 ± 0.12	KIN + 2,4-D	2.5 + 3.5	28
	67.04 ± 0.33	IAA + BAP	3.0 + 2.5	29
	67.59 ± 0.18	KIN + IAA	2.5 + 2.5	28
Kavya	70.20 ± 0.38	KIN	2.5	28
	71.31 ± 0.45	2,4-D	3.0	28
	68.42 ± 0.47	KIN + 2,4-D	2.5 + 3.5	29
	67.22 ± 0.25	IAA + BAP	3.0 + 2.5	30
	71.58 ± 0.36	KIN + IAA	2.5 + 2.5	29
Sambha mahsuri	78.49 ± 0.40	KIN	2.5	32
	80.28 ± 0.45	2,4-D	3.0	27
	79.21 ± 0.61	KIN + 2,4-D	2.5 + 3.5	29
	75.39 ± 0.52	IAA + BAP	3.0 + 2.5	29
	70.30 ± 0.67	KIN + IAA	2.5 + 2.5	28
Ramappa	65.48 ± 0.51	KIN	2.5	34
	64.37 ± 0.47	2,4-D	3.0	32
	61.48 ± 0.56	KIN + 2,4-D	2.5 + 3.5	32
	63.73 ± 0.51	IAA + BAP	3.0 + 2.5	31
	62.87 ± 0.13	KIN + IAA	2.5 + 2.5	30
Khushava	60.77 ± 0.24	KIN	2.5	30
	64.98 ± 0.28	2,4-D	3.0	29
	61.49 ± 0.32	KIN + 2,4-D	2.5 + 3.5	29
	59.30 ± 0.30	IAA + BAP	3.0 + 2.5	30
	63.57 ± 0.37	KIN + IAA	2.5 + 2.5	29

Those with best average values were considered in 21 varieties for example average response (%) below 50 % was not considered, below 8 multiple shoots were not considered and below 8 roots were not considered. Number of tubes per each concentration studied = 5; Number of replicas for each concentration studied = 2; Total number of tubes per each concentration studied = 5 × 2 = 10; Number of de-husked seeds used per tube = 1; Number of de-husked seeds used per concentration studied = 10

^a^ $${\text{Average response }}\left( \% \right) = \frac{{{\text{No of dehusked seeds showing response per concentration}} \; }}{\text{Total number of tubes per concentration}}\times \; 100$$

### Multiple shoot’s regeneration and root’s proliferation

Multiple shoots were achieved only in four cultivars (PR-116, PR-115, Sambha mahsuri and Erramallelu) out of 21 studied indica rice cultivars inoculated onto the MS culture medium. The proliferation of multiple shoots was optimized at different combinations with different concentrations of 2,4-D + kinetin (0.3 +2.1 mg L^−1^), BAP (5.0 mg L^−1^), 2,4-D + BAP (0.3 + 2.2 mg L^−1^), NAA + kinetin (2.0 + 2.1 mg L^−1^) and NAA + BAP (2.0 + 2.2 mg L^−1^), sucrose (3.0 %) and mannitol (1.0 %) (Table 2). Out of above mentioned cultivars, best multiple shoot proliferation (average number 114.51 ± 0.11 shoots) was observed in Sambha mahsuri when cultured onto BAP (5.0 mg L^−1^) (Fig. 1) similarly, moderate multiple shoot regeneration was recorded in PR-115 (average number 13.56 ± 0.36 shoots) at 5.0 mg L^−1^ of BAP (Table 2).

**Table 2:** Proliferation of multiple shoots in various cultivars of indica rice cultured on MS culture medium with various concentrations of growth regulators

Growth regulators	Concentration of growth regulators (mg L^−1^)	Number of days taken	Average number of shoots in selected cultivars^a^			
			*A*	*B*	*C*	*D*
MS	–	30	1	1	1	1
MS + BAP	5.0	27	13.56 ± 0.36	12.49 ± 0.42	114.51 ± 0.11	11.38 ± 0.48
MS + 2,4D + KIN	0.3 + 2.1	29	12.64 ± 0.65	11.58 ± 0.39	112.53 ± 0.30	10.55 ± 0.72
MS + 2,4D + BAP	0.3 + 2.2	27	10.26 ± 0.48	08.62 ± 0.35	13.43 ± 0.59	11.23 ± 0.63
MS + NAA + KIN	2.0 + 2.1	28	10.57 ± 0.49	11.93 ± 0.28	13.11 ± 0.39	10.39 ± 0.51
MS + NAA + BAP	2.0 + 2.2	29	10.84 ± 0.66	12.54 ± 0.49	12.39 ± 0.49	12.59 ± 0.42

(A) PR-115, (B) PR-116, (C) Sambha mahsuri, (D) Erramallelu

Number of tubes for each concentration was five, i.e., ten for each concentration studied

Seeds showing below eight shoots were not considered

Average of number of shoots per tube was calculated and mentioned in above table

Number of shoots: Number of shoots = (A1 + A2 + A3… + A10)^b^ = *X*; Number of tubes = *B*; Average of shoots = *X* ÷ *B*

*BAP* 6-Benzyl amino purine, *KIN* kinetin, *2,4-D* 2,4-Dichlorophenoxy acetic acid, *NAA* 1-Naphthaleneacetic acid

^a^Determination of shoots = Number of replicates for each concentration was two

^b^A1–A10 is number of test tubes

Profuse rooting was achieved in four cultivars of indica rice (PR- 116, PR-115, Sambha mahsuri and Erramallelu) when, cultured onto MS culture medium with supplementation of IBA (1.0 mg L^−1^), in 18–20 days, respectively (Table 3). The maximum rooting (average number 13.63 ± 0.83 roots per shoot) was achieved in Sambha mahsuri cultivar of indica rice with supplementation of IBA (1.0 mg L^−1^) after 18 days (Table 3; Fig. 1). Similarly, moderate rooting was also observed in Erramallelu (average number 10.44 ± 0.73 roots per shoot) cultivar of indica rice at concentration of IBA (1.0 mg L^−1^), in 19 days, respectively (Table 3). The in vitro regenerated plantlets after attaining a size of 2–3 inches were then taken out from aseptic containers and transferred to the natural conditions after acclimatization.

**Table 3:** Profuse rooting in multiple shoots of indica rice cultivars cultured on MS culture medium with various concentrations of growth regulators

Name of cultivars	Number of days	Average number of roots^a^	Growth regulator (IBA mg L^−1^)
PR-115	18	09.54 ± 0.48	1.0
PR-116	19	08.69 ± 0.68	1.0
Sambha mahsuri	18	13.63 ± 0.83	1.0
Erramallelu	19	10.44 ± 0.73	1.0

Number of replicates for each concentration was two

Number of tubes for each concentration was five, i.e., ten for each concentration studied

Shoots showing below 8 shoots were not considered

Average of number of roots per shoot in each tube was calculated and mentioned in above table

^a^Determination of roots = number of roots = (A1 + A2 + A3… + A10)^b^ = *X*; number of tubes = *B*; average of roots = *X* ÷ *B*

^b^A1–A10 is number of test tubes

## Discussion

In the current study, 21 cultivars of indica rice were used for micropropagation through de-husked seeds. The effects of 2,4-D were studied and observed that 2,4-D enhanced the frequency of induction and growth of callus (Deo et al. 2009). Similarly, in Sambha mahsuri cultivar, maximum multiple shoots regeneration was achieved in supplementation of MS + BAP (5.0 mg L^−1^). Simultaneously, stimulatory effects of BAP in combination with IAA and NAA have previously been reported to facilitate regeneration in indica rice cell cultures (Mandal et al. 2003; Karthekeyan et al. 2009). The observed results revealed that kinetin was found to be effective for the induction and proliferation of shoots and roots. This may be because cytokinin causes development of single pole and formation of meristematic dome from which the shoot primordium develops. The role of cytokinins on somatic embryogenesis has been explained by enhanced cell division of pre-embryogenically determined cells (Kintzois et al. 2002). In addition to these reports, kinetin increases proliferation and regeneration of callus by affecting mitosis, cytokinesis, total protein synthesis, lignin biosynthesis, vascular differentiation and differentiation of mature chloroplast from protoplastids (Wan et al. 1988).

An important aspect of the present protocol was that all the plantlets regenerated from mature embryo-derived calli developed good root system. Moreover, after transferring to pots, in vitro regenerated plantlets of four cultivars survived, which in turn indicated good adaptability of somaclones in ambient environmental conditions. High auxin concentration leads to development of root primordium leading to root development. However, one of our cultivar Sambha mahsuri decreases in root development at high auxin concentration. Thus, in turn, suggested that genotypes also possessed variability in their capability of regeneration as reported in previous studies (Kumaria et al. 2000). The present study establishes a successful protocol for the induction and growth of callus, somatic embryogenesis in four diverse cultivars (PR-115, PR-116, Sambha mahsuri, and Erramallelu) of Indian sub-continent, and the in vitro regenerated plants were showed yield potential at par to that of direct seeded plants in ambient conditions. The present protocol would be useful for genetic transformation in elite genotypes of rice using any selected gene transfer method in near future.

## Conclusion

Maximum frequency of induction and growth of callus, shoot proliferation was achieved in Sambha mahsuri cultivar of indica rice cultured onto MS culture medium with supplementation of 2,4-D (3.0 mg L^−1^) and BAP (5.0 mg L^−1^) in 27 days, while slow induction in other studied cultivars of indica rice. Thus, there is a need to develop new rice cultivars that could cope up to drought conditions and flourish into good quality of grain and higher yield. In our next course of the study, drought tolerant gene *DREB1A* will be introduced into Sambha mahsuri cultivar by particle bombardment method to improve of its growth and high yield under drought conditions for incremental benefits of farmers.
